# Improving Accessibility and Usability of Clinical Data for the Swiss Personalized Health Network: Development and Usability Study

**DOI:** 10.2196/86461

**Published:** 2026-08-11

**Authors:** Christophe Gaudet-Blavignac, Julien Ehrsam, Mirjam Mattei, Paloma Cito, Daniel Damian, Yves Jaggi, Jean-Louis Raisaro, Christian Lovis

**Affiliations:** 1 Division of Medical Information Sciences, Diagnostic Department, University Hospitals of Geneva, Rue Gabrielle-Perret-Gentil 4, Geneva, 1205, Switzerland, 41 223790815; 2 Department of Radiology and Medical Informatics, University of Geneva, Geneva, Switzerland; 3 Information Systems Directorate, Lausanne University Hospital, Lausanne, Switzerland; 4 Biomedical Data Science Center, Lausanne University Hospital and University of Lausanne, Lausanne, Switzerland

**Keywords:** semantic interoperability, knowledge representation, terminology standards, data models, ontology, SNOMED CT

## Abstract

**Background:**

In large-scale research initiatives such as the Swiss Personalized Health Network (SPHN), ensuring interoperability and ease of use across diverse clinical datasets is challenging due to a lack of standardization and semantics. The approach taken by the SPHN with the creation of a reference common dataset integrating various data structures and standards creates complexities for researchers aiming to explore and find specific clinical concepts to assess project feasibility. Semantic enrichment and exploration through SNOMED CT offer a potential solution by enabling structured queries that could simplify data discoverability and enhance dataset usability across Switzerland.

**Objective:**

This study evaluates how a semantic layer can improve the exploration of the SPHN dataset by leveraging SNOMED CT’s hierarchical terminology. To validate this hypothesis, we developed the Smart SNOMED Search for SPHN (S4) tool, which leverages a semantic enrichment of the dataset, facilitating semantic searches using the Expression Constraint Language of SNOMED CT.

**Methods:**

The SPHN dataset underwent semantic enrichment, where concepts and attributes not already represented were systematically mapped to SNOMED CT codes and associated value sets. An additional 717 meaning bindings and 232 value sets were created. The S4 tool was designed to enable Expression Constraint Language–based queries, allowing users to retrieve relevant SPHN concepts and value sets effectively. We tested the tool using a validation dataset representing commonly encountered clinical data warehouse elements and evaluated its precision, recall, and *F*_1_-scores.

**Results:**

The S4 tool demonstrated high accuracy, with an overall precision of 95.3%, recall of 97.5%, and an *F*_1_-score of 96.4%, indicating effective retrieval and alignment of SPHN concepts with SNOMED CT codes. The enrichment also highlighted gaps within the SPHN dataset, such as a lack of representation and the inability to use postcoordination, enabling enhanced semantic connections that further supported data discoverability.

**Conclusions:**

The S4 tool validates the hypothesis that semantic representation enhances data explorability within large frameworks such as the SPHN, making it more accessible for research. While effective, future work could address limitations by refining search precision and improving accessibility for users less familiar with SNOMED CT, thereby supporting SPHN’s mission to facilitate personalized health care research through enhanced data interoperability.

## Introduction

### Swiss Personalized Health Network

The Swiss Personalized Health Network (SPHN) is a national initiative, launched by the Swiss federal government in 2017 and run by the Swiss Academy of Medical Sciences to promote personalized medicine and health in Switzerland [1]. It contributes to the development, implementation, and validation of coordinated data infrastructures to help improve interoperability and reusability of clinical data for research in Switzerland. The SPHN has adopted a federative approach by building upon—and supporting—existing data sources and infrastructures across the country. It brings all decision-makers together to make health data interoperable and accessible for research.

### SPHN’s Semantic Strategy

To implement this vision, a strategy was defined to ensure the interoperability of research data among the projects supported by the initiative [2]. This strategy relies on 3 pillars. The first pillar, which this work builds upon, is the enforcement of a semantic representation of clinical data. International classifications exist for every type of data, and these semantic standards should be used whenever possible when representing data. No specific semantic standard is enforced, but SNOMED CT (SCT) is strongly recommended due to its inclusion in national recommendations for the semantic representation of clinical data and its advantages for searchability and explorability [3]. The second pillar is a model-agnostic data storage. The Resource Description Framework (RDF) has been chosen to hold this role so that semantically represented data can be stored in RDF structures regardless of the model. Finally, the third pillar of the strategy proposes creating ad hoc conversion scripts to transform and output data in any data model targeted for usage. These 3 pillars form the SPHN Semantic Interoperability Framework [4]. This framework is deployed by every participating institution across Switzerland including all 5 University Hospitals in the country. Every SPHN supported project must implement it, prompting the need for tools to support these implementations.

### The SPHN Dataset

To implement the first and second pillars of the strategy, a data schema of concepts, called the SPHN Dataset, is defined by the Data Coordination Center of SPHN and was first released in 2018 to which each hospital is asked to map its data which are then transformed in the RDF Turtle format [5]. Initially consisting of 8 core clinical concepts, it has since been expanded and improved iteratively through multiple releases, to currently consist of 165 concepts, most of them composed of multiple subconcepts named “attributes.” Attributes can have their values restricted to value sets or point to numeric, literal, or date-time values. The dataset is released with the derived RDF schema [6,7]. These concepts, attributes, and corresponding value sets are bound to international and national semantic standards. SCT was the main standard used, but depending on the concept, other international standards such as *ICD-10* (*International Classification of Diseases, Tenth Revision*), Anatomical Therapeutic Chemical, Global Trade Item Number, and others are also used.

The resulting dataset is complex and has proven challenging to adopt and implement by both hospital clinical data warehouses (CDWs) and researchers. The first challenge is the difficulty of searching through numerous SPHN concepts and attributes to understand which ones to use to represent data. Second, the variability of the classifications used has proven difficult to handle when exploring the dataset. Additionally, each new version of the dataset comes with modifications to concepts’ names, definitions, value sets, standards, and attributes, in addition to new concepts added with each release. This creates an additional workload each year to bring each hospital’s data representation up to date with the new release. Finally, each project that is part of the SPHN has the option of defining new concepts if the concepts in the current SPHN dataset release do not meet their needs [8]. This creates multiple parallel versions of the dataset, one for each project, with the possibility of one project reusing concepts from another, which can then also be added to the core dataset in a future release. A full understanding of how to use the dataset demands an in-depth knowledge and global view which only a few experts currently have. This is due partly to the fragmented nature of the health care system, with only a few people in each hospital in the SPHN initiative knowledgeable about the entire structure. The setup of the SPHN network is such that these experts are located throughout the country, and each university hospital has a few, but the sustainability of such an approach is questionable.

### Related Work

In similar systems such as the Observational Medical Outcomes Partnership or Informatics for Integrating Biology and the Bedside, tools are available to browse through the concepts covered. Informatics for Integrating Biology and the Bedside has a concept search bar allowing text search and a tree-based interface to explore concepts hierarchically [9]. The Observational Medical Outcomes Partnership has a terminology server named Athena, allowing text search and navigation into related concepts [10]. Although similar work has explored the use of SCT for information extraction and cohort identification, tools to support users are lacking for the SPHN [11,12]. Moreover, to the best of our knowledge, none of these systems include semantic search using a formal language such as the Expression Constraint Language (ECL) of SCT. This capability would provide clear advantages over a simple textual search by using hierarchical relations, synonyms, and logical definitions as is possible in various available SCT browsers [13].

**Figure 1.**
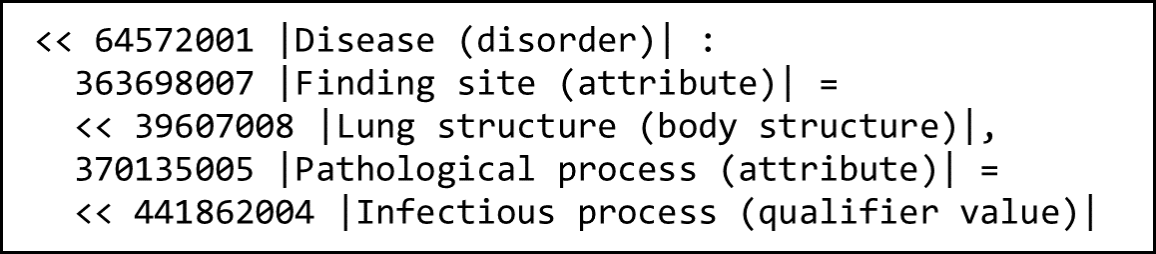
Example of an Expression Constraint Language (ECL) query representing a disease located in the lung and including an infectious process.

The ECL can be used to compose queries using SCT concepts, combining them with a specific syntax, to gather a relevant set of concepts matching the query. This language can be used to express clinical meanings such as “disorder of the lung,” “procedure measuring creatinine,” and “body parts included in the lower limb,” thus enabling a standardized concept exploration language across data models and tools. Snowstorm, the terminology server provided by SNOMED International, can parse those queries and retrieve relevant codes with a simple application programming interface call [14]. The example query shown in Figure 1, when submitted to a Snowstorm server, will output a set of 270 SCT concepts from the International Edition September 2024 version that are lung infections, such as “882784691000119100 |Pneumonia caused by severe acute respiratory syndrome coronavirus 2 (disorder)|” or “10625311000119109 |Bronchopneumonia caused by Klebsiella pneumoniae (disorder)|.” This semantic query language can be used to search through SCT-represented data in a meaningful way. Given that the majority of the SPHN dataset is represented using SCT, we aimed to find out whether the semantic content embedded in SCT could facilitate searching through SPHN concepts and value sets.

This paper proposes a novel approach to enhance interaction with the SPHN dataset by leveraging the semantic capabilities of SCT. Specifically, we aim to investigate whether the integration of a robust semantic layer based on SCT can facilitate the exploration of a clinical dataset within a national research framework, either by improving concept findability, or by reducing the time needed to find them. Our approach builds upon our team’s extensive expertise in both SPHN and SCT, which enabled us to develop the Smart SNOMED Search for SPHN (S4) tool. This tool is designed to leverage the use of SCT within the SPHN dataset to facilitate semantic queries, reduce manual effort, and ultimately support interoperability to improve the accessibility and usability of the dataset.

## Methods

### Dataset Semantic Enrichment

In the SPHN dataset, a concept has a name, a description, a meaning binding that acts as the link with a controlled vocabulary to support machine readability, and one or several attributes called “composedOfs” (Table 1). A concept can be seen as a “Class” in object-oriented programming. The attributes also have a name and a description, but no meaning binding. They can, however, have value sets or literals of type temporal, string, or Quantity. Concepts do not have value sets, and actual clinical data are instantiated through attributes and not concepts. Note that, in SPHN, only attributes of type “Code” can have value sets expressed in a terminology system such as SCT; other attributes do not.

An in-depth gap analysis, based on an established semantic representation framework, of the current dataset’s concepts, attributes, definitions, and value sets was performed to determine exactly which ones were deemed to be insufficiently or inappropriately represented by SCT [15]. These concepts and their attributes require semantic enrichment to cover a maximum of relevant data with SCT expressions. The semantic enrichment can be achieved by adapting the SPHN concept in 2 different ways. First, the terminology code used to define the concept (referred to as “meaning binding” hereafter) can either be modified or completed if absent. Second, the value set can also either be improved when deemed incomplete or created when SCT was not present. During this enrichment process, the structure of the SPHN dataset is not altered. As clinical information is also present in attributes that are not of type “Code,” it was decided to semantically enrich their value sets when possible and relevant (even though the data is not instantiated directly using these semantic codes). As such, the semantic enrichment is applied to both the meaning bindings and the value sets. This is done for the 2 most recent versions of the dataset, resulting in 2 new semantically augmented versions of the dataset, the 2023 and 2024 releases. This approach was taken as work started on the 2023 version but was spread to the 2024 upon its release, as it contained many new concepts that also needed to be included.

**Table 1.** An example of the instantiation of clinical data for the concept “Lab Test,” with all the information present in the attributes.

Concept	Lab test	Value
composedOf	code	2951‐2 (Sodium [Moles/volume] in Serum or Plasma)
composedOf	instrument	04015630930845 (cobas 8000 c 702 Module)
composedOf	test kit	08430215011546 (Sodium Electrode)
composedOf	result	140 mmol/L

### Search Algorithm

**Figure 2.**
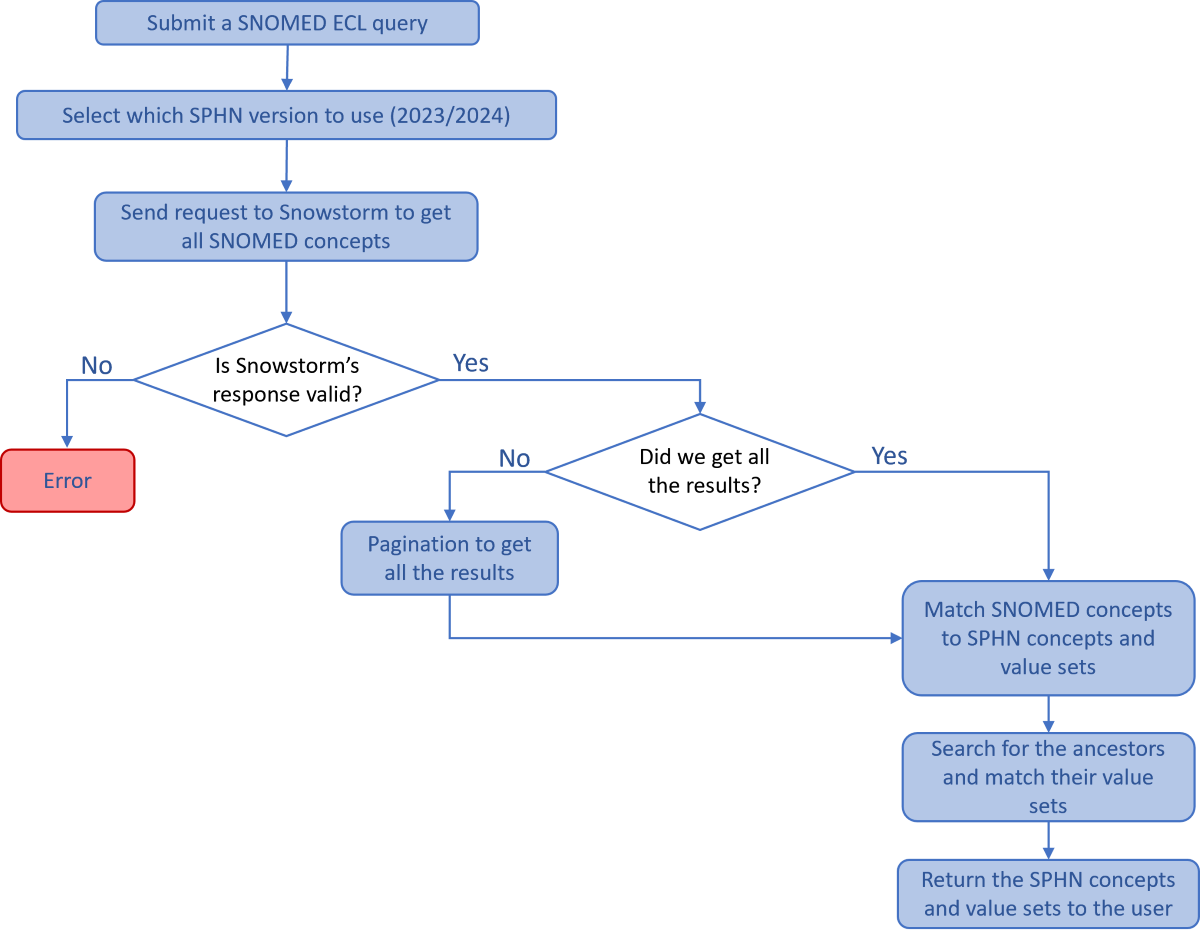
Handling of a query in the app. ECL: Expression Constraint Language; SPHN: Swiss Personalized Health Network.

**Figure 3.**
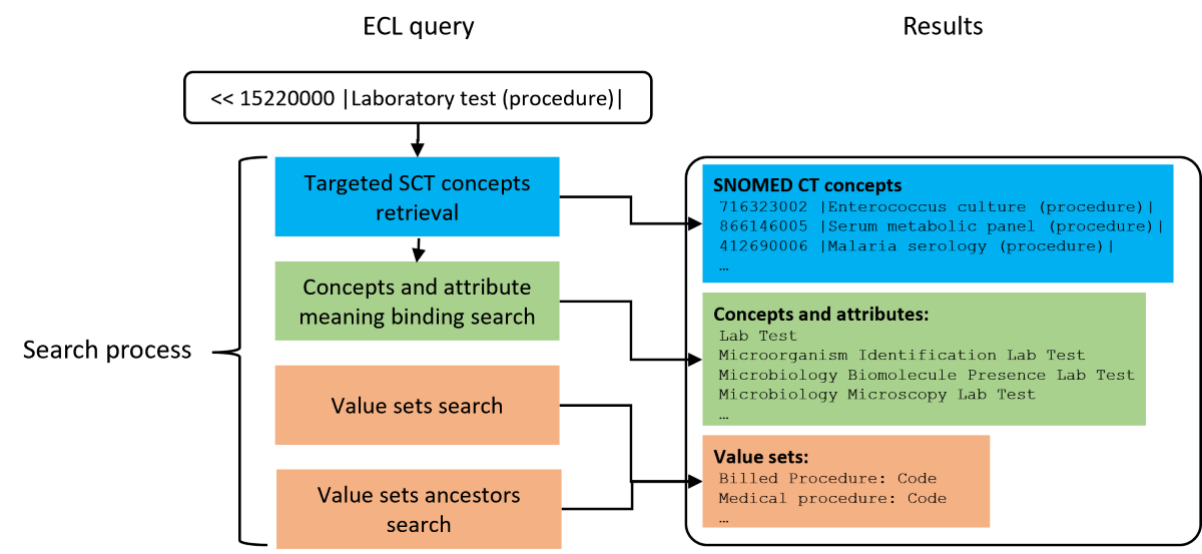
Example of a query process and its results. ECL: Expression Constraint Language; SCT: SNOMED CT.

The algorithm developed to search in the SPHN dataset from an ECL query considers the specific design of the dataset. It can be described as follows: the ECL query is sent to the Snowstorm application programming interface to retrieve all SCT concepts targeted by the query. For example, the query “<<50373000 |Body height measure (observable entity)|” will return the Body height measure code and all its 18 descendants in the September 2024 version of the International Edition of SCT. These are then compared to 2 different datasets. The first version, the concept dataset, links SCT concepts to SPHN concepts or attributes. The second version, the value set dataset, links SCT concepts to SPHN value sets that could include them. For each SCT concept returned by the query, the set of corresponding SPHN concepts and attributes is retrieved from the first dataset and added to the results. Then, each targeted SCT concept is searched in the second dataset and added to the results in a “value set section.” Certain value sets are defined as all descendants of a concept (eg, “descendant of: 117259009 |Microscopy (procedure)|”) which means that all children of this concept are included in the value set. For these cases, the ancestors of each concept resulting from the query are retrieved in Snowstorm and matched again to the same dataset, ensuring that no ancestors are missed. Searching using all the descendants also ensures that all the relevant SPHN concepts can be found. Finally, the SPHN concepts retrieved are added to the aggregated results. The process of the app is displayed in Figure 2, and an example is provided in Figure 3. The benefit of this search method is that it allows the results to be presented in a clear way, pointing exactly to where in the dataset the queried information lies. This facilitates the exploration and usability of the dataset, as there is no longer a need to manually explore it to find the relevant information.

### App Design

**Figure 4.**
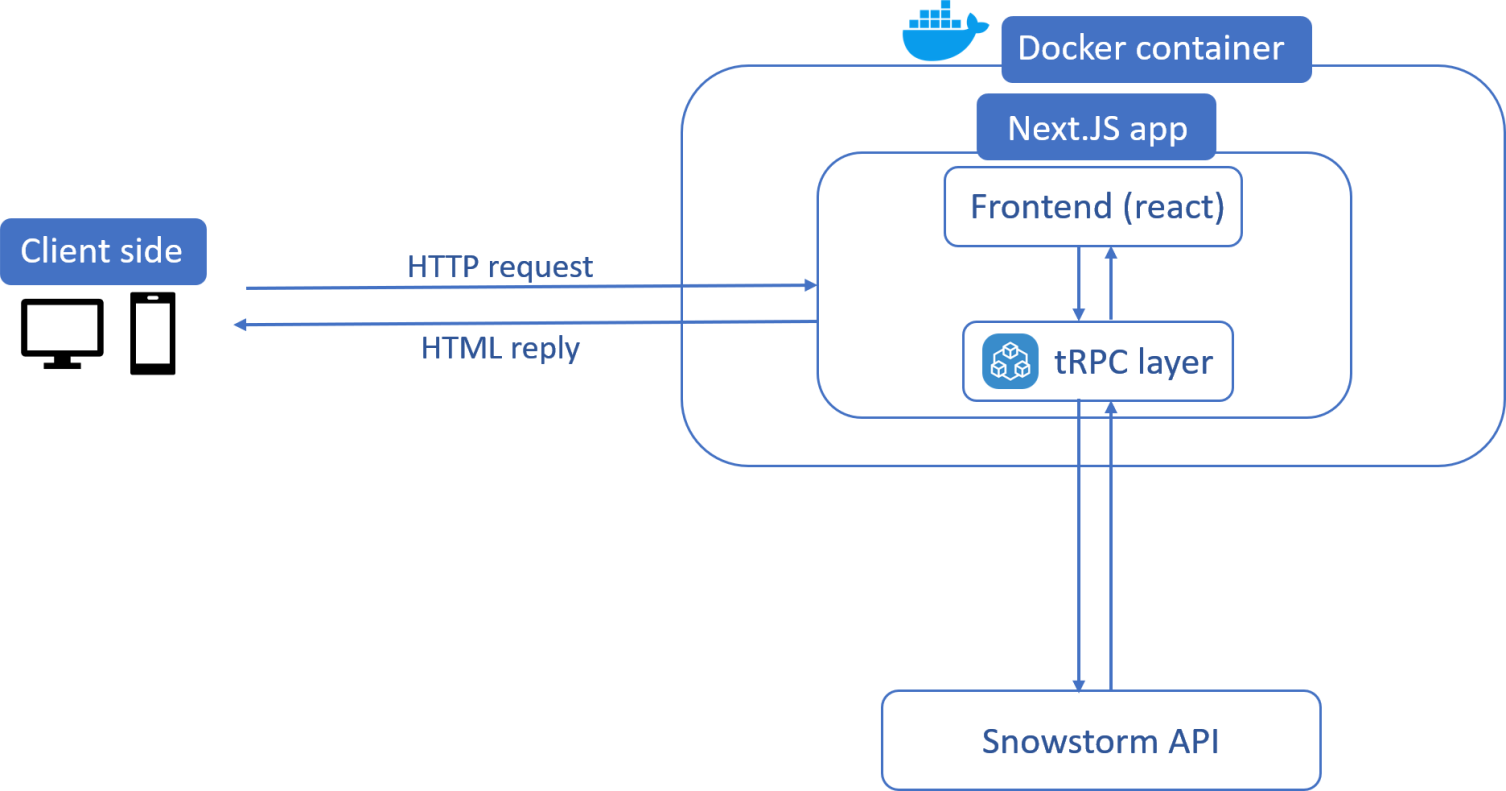
Architecture of the S4 system. API: application programming interface; HTTP: Hypertext Transfer Protocol; tRPC: TypeScript Remote Procedure Call.

**Figure 5.**
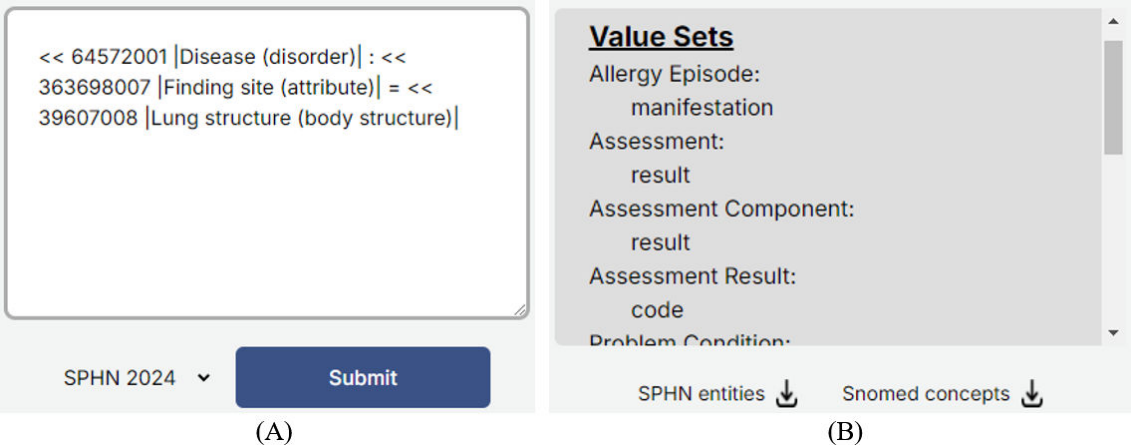
(A) Search box in the web app. (B) Results of an Expression Constraint Language (ECL) query. SPHN: Swiss Personalized Health Network.

**Figure 6.**
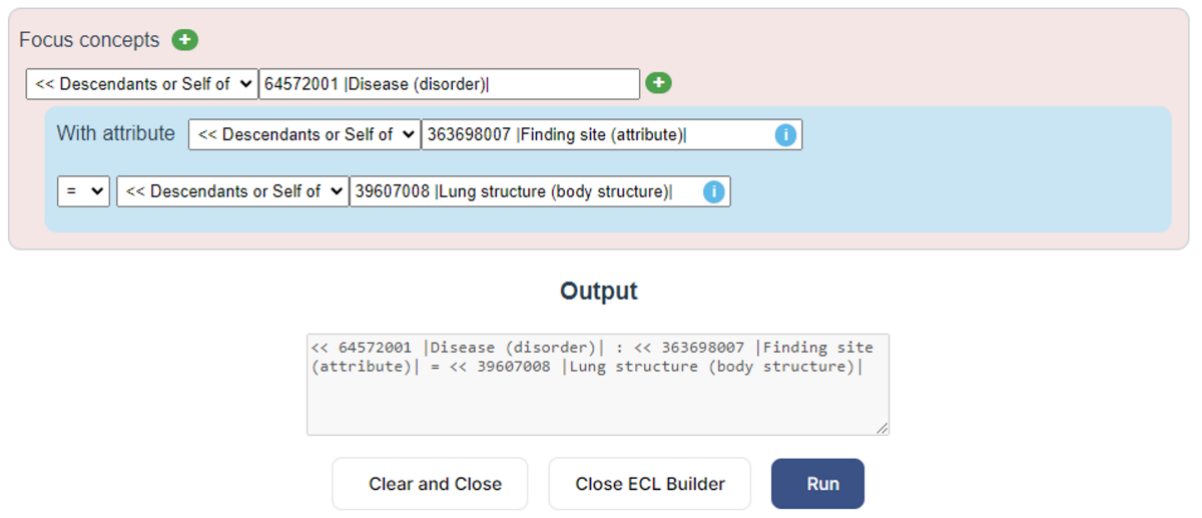
Expression Constraint Language (ECL) builder for easy query creation.

A web app has been developed to support users willing to explore the SPHN dataset in more detail using semantic queries. It is developed using the React framework Next.js [16]. The app is bundled in a Docker container and is configured to interact with an instance of Snowstorm (Release 10.4.2 containing the September 2024 release of the SCT International Edition), the terminology server provided by SNOMED International (Figure 4) [14]. The frontend provides 2 ways of creating an ECL query correctly, ensuring semantically and syntactically accurate queries that are indispensable for subsequent analyses. First, a search box is shown, in which one can type out or paste an ECL query (Figure 5A). Second, a query builder, similar to the one available in the SNOMED Browser [17], is also provided for less experienced users. From here, an ECL query can be rapidly created in a stepwise and guided manner and used to launch a query (Figure 6).

Once the query has been defined, the search algorithm is executed to match the SCT concepts targeted by the ECL to the enriched SPHN dataset. The primary output consists of the SPHN concepts and value sets returned, specifying whether they are part of a meaning binding or a value set. These results are displayed to the user and are available for download in comma-separated values format to allow easy manipulation (Figure 5B). The results also display the number of SCT concepts that match the query, and these can also be downloaded.

### Evaluation

A validation dataset is defined to evaluate the method’s performance. It is composed of a set of frequently occurring data elements chosen from categorical data from one of the participating hospitals’ CDW that has been previously manually represented using SCT expressions [15]. They are selected empirically to cover a broad range of clinical data from various sources such as laboratory procedures, clinical scales, medical devices, radiology, observations, drugs, diagnoses, and others. In order to best represent the semantic space of the SPHN dataset and ensure the evaluation is representative of the task, the 20 most frequent data elements were selected from each source to be used for validation. This means the validation set contains both a broad range of data sources, and a sufficient number of each to ensure they are relevant. Then, only data elements that are deemed relevant for research are kept, with elements such as form names removed, as they are not present in the SPHN dataset and therefore cannot be used for research in the SPHN context. Expressions, both simple and postcoordinated, are then reviewed to ensure correctness and compliance with the SNOMED compositional grammar.

The result of this process is a total of 438 SCT codes and expressions. The SCT expressions representing the data elements were then transformed into ECL queries with equivalent meanings. The transformation was aimed at converting syntactic elements authorized for postcoordination into corresponding elements in ECL. Pragmatic modifications were also made to better imitate the way a user would search the SPHN dataset, including the addition of subtype or supertype modifiers (<, <<, >, >>), and the modification of coordination signs (“+” into “,”) for simple multiple focus expressions [18].

The resulting SCT codes and expressions were manually evaluated by 2 experts in SCT and the SPHN dataset to determine in which SPHN concept or value set the information corresponding to the SCT expressions should be included. The differences between the 2 experts’ evaluations were then discussed between them and an additional supervising expert, when needed, until consensus was reached on all points. This final expert evaluation set then serves as the gold standard. The list is then submitted to the software and the resulting SPHN concepts and value sets are compared with the gold standard. Metrics such as precision, recall, and *F*_1_-score are computed to evaluate the validity of the software.

## Results

### Deployment Validation

The final app was deployed in 2 Swiss university hospitals and in the SPHN-dedicated environment of another research project to ensure its compatibility and ease of installation. The architecture of the tool was based on Docker technology to reduce configuration and deployment complications. For this, the B-Space of the LUCID project was used. This space consisted of a 64-bit server running Red Hat Enterprise Linux (RHEL) version 8.10, operating on a 4.18.0 Linux kernel. The LUCID project is an NDS SPHN project focused on low-value care [19]. The tool was installed, deployed, and used by the LUCID team. Although no formal evaluation was conducted, no portability, configuration, or integration issues were raised. The app is published as open source on a GitHub repository [20].

### Semantic Enrichment

The semantic enrichment was done for both meaning bindings and value sets, for the 2023 and 2024 versions of the SPHN dataset to improve the findability of information and the reusability of the dataset. The in-depth analysis of the modifications and differences with the SPHN dataset is described below for the 2024 version, as it is the most recent and complete version of the dataset. However, both enriched S4 datasets can be queried on the S4 tool. The complete 2024 enriched dataset is available on GitHub as well [20].

**Figure 7.**
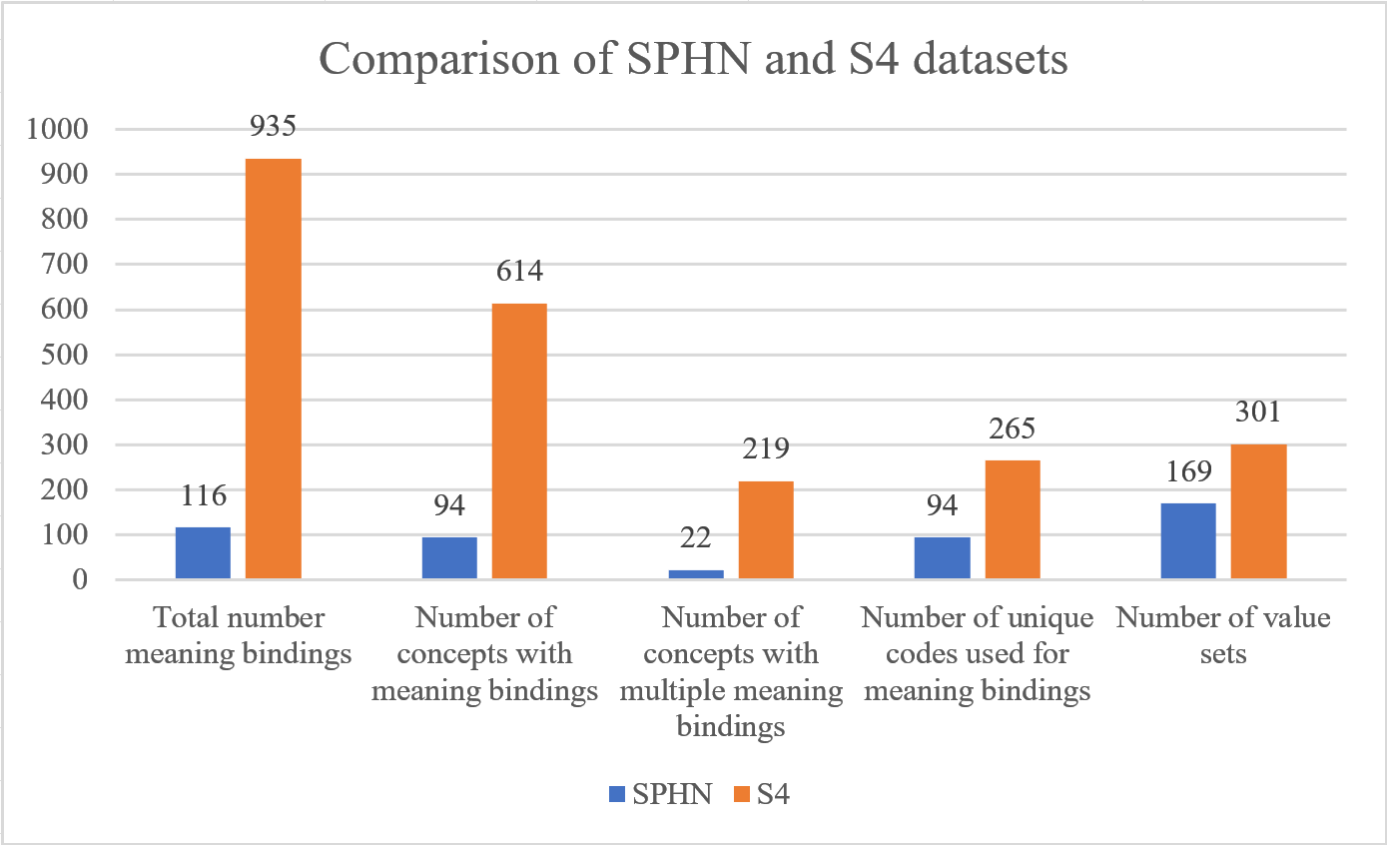
Comparison of the Swiss Personalized Health Network (SPHN) dataset and the semantically enhanced S4 dataset.

In the final dataset, a total of 614 concepts and attributes (75% of the total 820 concepts and attributes) were enriched with 935 SCT meaning bindings compared with 77 (9%) before enrichment, representing a total of 265 distinct meaning bindings (Figure 7). Of the 77 already in SNOMED CT, 72 were considered correct and not modified, and 5 were deemed insufficient and augmented. When relevant, new value sets were added to the existing ones. Similarly, some value sets that were defined in standards other than SCT were added as SCT value sets, and others were added where there was no specified standard to be used. In total, the number of attributes with defined value sets increased from 169 in the SPHN dataset to 301 in the enriched dataset, including 189 that previously did not have one, and 43 that were modified.

Multiple meaning bindings were assigned to single concepts or attributes to increase the chance of finding an SPHN concept using SCT codes. For example, the body temperature result attribute of the Body Temperature Measurement concept can be found via either the 105723007 |Body temperature finding (finding)| or 386725007 |Body temperature (observable entity) | SCT concepts. This ensures that the concept in question can be found by searching for both the question (observable entity) and the answer (finding). Therefore, a greater number of SCT codes were used for the meaning binding compared to the original dataset. This addition was only made when the semantic representation was deemed correct to maintain a reproducible and coherent mapping throughout the project. Additionally, we extended meaning bindings to attributes, whereas SPHN currently only provides them for concepts. This is due to the structure of the SPHN, which can reuse a concept as an attribute in another concept, slightly changing its meaning. We deemed it important to also assign these attributes with a meaning binding, while keeping them in the context of their concept. For example, the “age quantity” attribute is used in 2 concepts, “Age” and “Gestational Age At Birth.” These have slightly different meanings and are therefore assigned different meaning bindings, “424144002 |Current chronological age (observable entity)|” and “412726003 |Length of gestation at birth (observable entity)|,” respectively. Concepts or attributes without a meaning binding (200) and attributes without a value set (354) either had no clinical value (datetimes), no semantic value (technical concepts), or simply no adequate representation available in SCT. These have been maintained in the S4 dataset for clarity but cannot be found using the tool. Since these have no clinical or semantic value, this was not deemed to affect the use of the tool in any significant manner. All SPHN meaning bindings and value sets have also been maintained, ensuring findability for users who are already familiar with the SPHN dataset (Table 2). A comparison of the SPHN and S4 datasets is provided in Figure 7. The complete enriched dataset is available on the GitHub repository [20].

**Table 2.** Comparison of the Swiss Personalized Health Network (SPHN) dataset and the semantically enhanced S4 dataset.

Category	SPHN (version 2024.2)	Enriched dataset
Number of concepts/attributes (N=820)		
With meaning binding	94 (77 SCT^a^)	614
With multiple meaning bindings	22	219
Number of unique meaning bindings	94 (77 SCT)	265
Number of attributes with defined value sets	169 (93 SCT)	301
Added	—^b^	189
Modified	—	43

a SCT: SNOMED CT.

b Not applicable.

### Evaluation

A formal evaluation was conducted for the 2024 version, as it was the most recent and complete one. It also covers everything the 2023 version covered, as there were only additions and modifications made, so there was no need to evaluate both separately. Two gold standards were created separately by both expert reviewers and compared by an interannotator agreement study. The agreement rate was high with a precision of 93%, recall of 95%, and an *F*_1_-score of 94% for one expert to the other. Discussion of differences based on semantic precision, through rounds of debate led by a third expert, led to complete agreement, and a gold standard was established. The gold standard was programmatically submitted to the app, and SPHN concepts and value sets were retrieved and stored in a separate file. This output was then compared to the gold standard. The results showed an overall precision of 95.3%, a recall of 97.5%, and an *F*_1_-score of 96.4%, underscoring the accuracy and reliability of the solution. The results of the evaluation are summarized in Table 3.

**Table 3.** Results of the evaluation.

Metric	Meaning bindings (%)	Value sets (%)	Total (%)
Precision	99.00	95.12	95.32
Recall	95.21	97.60	97.47
*F*_1_-score	97.07	96.34	96.38

## Discussion

### Principal Findings

This work highlights the potential of a semantic-driven approach to support data FAIRness in a national research data framework. Specifically, S4 semantically enriches the SPHN dataset by expanding its SCT meaning bindings and refining value sets, making clinical information more findable for CDW teams and reusable for researchers. By allowing researchers to use structured semantic searches through ECL queries to find which part of the SPHN dataset corresponds to their needs, the S4 tool improves data reusability and interoperability across Swiss hospitals. For instance, a researcher investigating complications related to central venous catheters can use S4 to instantly identify all relevant attributes and value sets. By submitting an ECL query targeting this device, the tool not only identifies the “Medical Device” concept but also precisely directs the user to associated “Procedure” attributes where these devices are specified, bypassing a tedious manual exploration of the complex SPHN data structures.

During the enrichment process, several gaps were identified in the SPHN dataset, prompting the addition of new SCT mappings. Several concepts or value sets that could have been represented using SCT were represented using other terminologies or lacked any representation. Value sets were sometimes defined using a closed set of strings, preventing any semantic processing on them. These gaps diminished the findability of the concepts and prevented the use of the SCT structure for queries. Filling those gaps allowed the creation of a powerful tool to search through this dataset, and this enrichment has been made public, to allow its implementation in subsequent updates of the dataset.

One of the key issues in the enrichment of the dataset was the fact that the SPHN framework does not allow postcoordination of SCT concepts. Postcoordination is the composition of multiple concepts according to a set of rules (named compositional grammar). This is one of the key features of SCT and allows the extreme representability of this language with a limited set of concepts. Some meaning bindings or value sets could not be fully represented without combinations of concepts, which are not allowed in SPHN, highlighting the usefulness of compositional terminologies to maximize semantic representation.

The evaluation set was conceived to represent data elements found frequently in a clinical data warehouse. This choice was made because the SPHN framework was created to leverage research by allowing more efficient data sharing across institutions in Switzerland. Most of the projects funded by the initiative were using health care data extracted from CDWs. By using the most frequent elements found in a CDW, we created a realistic set of elements that would be included in a research project with high likelihood. The results of the evaluation showed that the semantic bridge created between SCT representation and the SPHN dataset was working adequately.

### Limitations

There are a few limitations to this work. The main limitation is that as this tool is made specifically for exploring the SPHN dataset using SCT, it is aimed at a restricted set of people who are familiar with SCT and use the SPHN. Indeed, users need to know how to either write out a full query, create one using the ECL builder, or use the SNOMED Browser to retrieve codes. To mitigate this, we have created a user guide in the form of a tutorial for using the tool. Available on the tool’s home page and in the repository, it guides the user through an example to query the dataset using various types of input: single code, simple query, and ECL builder. It also explains how the results should be interpreted. The hope is that, with such a guide in place, the tool can be more accessible to a wider audience. As a future research direction, enabling accurate translation between natural language and SCT or ECL language by using large language models to translate free-text queries into ECL queries could significantly enhance accessibility and usability for a broader range of users.

Another limitation we have encountered is the fact that expanding the value sets and meaning bindings, as we have done means that in some cases, we get more results than necessary. Some of these are outright incorrect; for example, the query “<<302497006 |Hemodialysis (procedure)|” will return the correct SPHN concept “Billed procedure-code” but also the incorrect “Measurement-measurement method code.” This is because the value set defined by SPHN for the measurement method includes all descendants of the “128927009 |Procedure by method (procedure)|” code, which includes Hemodialysis, despite Hemodialysis obviously not being a type of measurement method. These discrepancies lie in inaccurate meaning bindings inherited from the SPHN dataset and could be used to improve the dataset. While those results are not necessarily false, they are sometimes too semantically distant from the intended query to be clinically relevant. One of the reasons we undertook this work is that the current semantic representation of the SPHN dataset was incomplete (Table 2), and without extensive knowledge of its creation process, it was impossible to search through it using semantic standards to find something specific. Using only the existing value sets and meaning bindings would not have permitted any further exploration. Therefore, augmenting the search results was part of the motivation. While this approach may result in an abundance of results, requiring additional filtering, we prioritized ensuring comprehensive coverage over the risk of missing relevant information.

### Conclusions

The S4 app takes a significant step forward in enhancing the accessibility and usability of clinical data for researchers, aligning seamlessly with the broader goals of the Swiss Personalized Health Network. This foundational improvement lays the groundwork for more integrated and advanced data exploration, crucial in supporting personalized health care research.

## Abbreviations

CDW: clinical data warehouse
ECL: Expression Constraint Language
*ICD-10*: *International Classification of Diseases, Tenth Revision*
RDF: Resource Description Framework
SCT: SNOMED CT
SPHN: Swiss Personalized Health Network
